# New insights into the mechanisms of high‐fat diet mediated gut microbiota in chronic diseases

**DOI:** 10.1002/imt2.69

**Published:** 2023-01-05

**Authors:** Jiali Chen, Yuhang Xiao, Dongmei Li, Shiqing Zhang, Yingzi Wu, Qing Zhang, Weibin Bai

**Affiliations:** ^1^ Department of Food Science and Engineering, Institute of Food Safety and Nutrition, Guangdong Engineering Technology Center of Food Safety Molecular Rapid Detection Jinan University Guangzhou China; ^2^ School of Chinese Medicine, Centre for Cancer and Inflammation Research Hong Kong Baptist University Hong Kong China; ^3^ Department of Microbiology & Immunology Georgetown University Medical Center Washington District of Columbia USA; ^4^ JNU‐HKUST Joint Laboratory for Neuroscience and Innovative Drug Research, College of Pharmacy Jinan University Guangzhou China

**Keywords:** characteristic metabolites, chronic diseases, gut microbiota dysbiosis, high‐fat diet, targeted biomarkers

## Abstract

High‐fat diet (HFD) has been recognized as a primary factor in the risk of chronic disease. Obesity, diabetes, gastrointestinal diseases, neurodegenerative diseases, and cardiovascular diseases have long been known as chronic diseases with high worldwide incidence. In this review, the influences of gut microbiota and their corresponding bacterial metabolites on the mechanisms of HFD‐induced chronic diseases are systematically summarized. Gut microbiota imbalance is also known to increase susceptibility to diseases. Several studies have proven that HFD has a negative impact on gut microbiota, also exacerbating the course of many chronic diseases through increased populations of *Erysipelotrichaceae*, facultative anaerobic bacteria, and opportunistic pathogens. Since bile acids, lipopolysaccharide, short‐chain fatty acids, and trimethylamine *N*‐oxide have long been known as common features of bacterial metabolites, we will explore the possibility of synergistic mechanisms among those metabolites and gut microbiota in the context of HFD‐induced chronic diseases. Recent literature concerning the mechanistic actions of HFD‐mediated gut microbiota have been collected from PubMed, Google Scholar, and Scopus. The aim of this review is to provide new insights into those mechanisms and to point out the potential biomarkers of HFD‐mediated gut microbiota.

## Highlights

- Gut microbiota dysbiosis increases the host's susceptibility to diseases.
- Identified characteristic microbes in high‐fat diet (HFD) induced obesity, diabetes, gastrointestinal diseases, neurodegeneration, and cardiovascular diseases.
- Bile acids, lipopolysaccharide, short‐chain fatty acids, and trimethylamine *N*‐oxide are the commonalities in HFD‐induced chronic diseases.
- FXR, TGR5, NF‐κB, PPAR‐*γ*, and PERK signaling were critical for the interacted mechanisms toward microbiota modulation.
- Provided new insights into mechanisms among microbiota, metabolites, and immune responses in chronic diseases.

## Graphical abstract

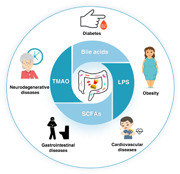

## INTRODUCTION

Daily food intake is one of the most effective and common ways for humans to access nutrition. However, excessive consumption of saturated fat and trans‐fatty acid from food may accompany with a series of chronic diseases. Nowadays, rapid development of food manufacturing industry has led to the changes of human lifestyle and dietary patterns, especially for the popularity of high‐fat diet (HFD) [1, 2]. It is known that the typical American (‘Western’) diet contains about 36 to 40 percent fat, with ‘tolerable’ high‐fat diets allowing as much as 50 to 60 percent energy from fat [3]. However, a 60% fat diet for rodents will not necessarily produce results likely to align well with human studies, where the usual ‘high‐fat diet’ for humans ranges from 52 to 60 % energy from fat [4, 5, 6]. It has been suggested that a better correlation will be achieved using a 45–60% fat diet for rodents [3, 7]. Numerous studies have revealed that HFD possessed a negative correlation in human health, including weight gain, organ fat accumulation, gut microbiota dysbiosis, insulin resistance, colonic injury, oxidative stress, cognitive impairment [8, 9, 10, 11]. Chronic diseases are the leading cause of death with 70% mortality [12]. Fanelli et al. [13] found that most patients with chronic disease had specific dietary characteristics, including excessive saturated fat intake, low total carbohydrate, fiber intake, and excessive sugar consumption. Raising evidence has demonstrated that HFD can be treated as the risk factor for chronic diseases, which should be of high concern [13].

Gut microbiota is the general designation of the microorganism community in the human gastrointestinal tract. It contains more than 1000 bacterial species with 10^13^–10^14^ bacteria [12, 14]. Several studies have noted a negative correlation between gut microbiota dysbiosis and host health [15, 16]. Dysbiosis is defined as a condition characterized by an unbalanced intestinal microbial community. Numerous research reported that gut microbiota dysbiosis may deliver a profound negative impact on the aggravation of chronic diseases, such as obesity, diabetes, cardiovascular diseases (CVDs), gastrointestinal diseases, and central nervous system disorders [17, 18, 19, 20]. Additionally, the composition of gut microbiota could be influenced by various potential factors, including long‐term lifestyle changes, diet, nutrition, pharmacological factors, infection, pregnancy, and host genetic factors [21, 22, 23, 24, 25, 26, 27]. Accordingly, the gut microbial community plays a pivotal role in the course of chronic diseases induced by the high‐fat and high‐sugar diet (HF/HSD). It is imperative to further summarize the data linking among HFDs, gut microbiota, and chronic diseases.

To date, numerous reviews have focused on the discussion of the changes of gut microbiota mediated by HFD based on certain diseases [28, 29, 30, 31, 32, 33]. Murphy et al. [34] indicated that intestinal bacteria was a risk factor for chronic diseases induced by HFD. Western diet is generally characterized as HFD with low‐fiber nutrition. Shi also emphasized that intestinal bacteria were an important link between the western diet and chronic diseases [35]. However, the current review remains with a lack of discussion of the latest research on the changes in gut dysbiosis induced by HFD and its corresponding mechanisms in major chronic diseases. Hence, this manuscript aims to systematically summarize the characterization of gut microbiota in various chronic diseases and revealed the potential interacted mechanisms in HFD pattern. The following sections systematically illustrated the influences of gut microbiota and its corresponding mechanisms under five popular chronic diseases, including obesity, diabetes, CVDs, gastrointestinal diseases, and neurodegenerative diseases.

### Influences of HFD on gut microbiota toward obesity

Obesity was first defined as a chronic disease by WHO in 1977. In 2016, worldwide incidence of obesity had already reached 13% [36]. It may be caused by the excessive intake of energy‐rich foods with high‐fat constituents [37]. Accumulating evidence also indicated that the dysbiosis of gut microbiota plays a major role in the pathogenesis of diet‐induced obesity. Clinical studies have indicated that the alteration of the Firmicutes/Bacteroidetes ratio (F/B ratio) might be closely linked to the occurrence of obesity [38]. Ley et al. [39] also found that people with obesity exhibited a relatively lower abundance of Bacteroidetes. Additionally, HFD model animal trials are commonly applied for evaluating the relevance of gut microbiota in diet‐induced obesity (Table 1) [10]. Notely, Bäckhed et al. [61] revealed that obesity could not be triggered by HF/HSD in germ‐free mice compared with normal mice.

**Table 1:** HFD mediated gut microbiota alterations among obesity, diabetes, cardiovascular diseases, gastrointestinal diseases, and neurodegenerative diseases.

Chronic diseases	Model	Diet	Method	Gut microbiota					Metabolites	References
				Firmicutes	Bacteroidetes	Proteobacteria	Actinobacteria	Others		
Obesity	Human	\	16S rRNA	↑Firmicutes	↓Bacteroidetes	\	\	\	\	[39]
Obesity	Germ‐free male C57BL/6J mice	41% fat; 42.7% sugar (4 weeks)	Shotgun Sequencing	↑Firmicutes ↑*Eubacterium dolichum*	↑Bacteroidetes ↓Bacteroidales	↑Proteobacteria	\	↑*Mollicutes*	↑Acetate ↑Butyrate ↑Lactate	[10]
Obesity	Male C57BL/6NCrl mice	60% fat (12 weeks)	16S rRNA	↑*Lactobacilli* ↑Erysipelotrichales ↓Clostridiales ↓*Ruminococcaceae*	↑*Rikenellaceae*	\	\	\	\	[40]
Obesity	Male Wistar rats	45% fat (6 weeks)	16S rRNA	↑F/B ↑The Clostridiales order (family *Clostrideacea* and RC4‐4)	↑*Butyricimonas*	↑unclassified RF32 ↑*Desulfovibrionaceae*	\	↓Archaea ↑*Deferribacteres* ↑*Cyanobacteria* ↑*Corynebacterium*	\	[41]
Obesity	Male C57BL/6N mice	48% fat (4 weeks)	16S rRNA	↑Clostridiales ↑*Acetatifactor muris* ↑*Eubacterium coprostanoligenes*	↓Bacteroidales	\	\	\	\	[42]
Obesity	Male C57BL/6J	45% fat (12 weeks)	16S rRNA	↓*Ruminococcaceae*	\	\	\	↑S24_7 ↓SCFA bacteria	Butyrate	[43]
Obesity	Male rats	60% fat (8 weeks)	16S rRNA	↑Firmicutes ↑F/B ↑*Ruminococcaceae* ↑*Erysipelotrichaceae* ↑*Christensenellaceae* ↓*Veillonellaceae*	↓Bacteroidetes ↓*Prevotellaceae* ↓*Bacteroides*	↑Proteobacteria ↑*Desulfovibrionaceae*	\	\	↓Acetic acid ↓Butyric acid ↓Pentanoic acid	[44]
Obesity	Male C57BL/6J mice	60% fat (8 weeks)	16S rRNA	↑Firmicutes ↑*Oscillospira* ↑*Ruminococcus* ↑Clostridiales	↓Bacteroidetes ↓Bacteria S24‐7 ↓Bacteroidales	↑Proteobacteria	↓Actinobacteria	\	\	[11]
Diabetes	Male C57bl6/J mice	72% fat (13 weeks)	In situ hybridization analysis	↓*Eubacterium rectale–Clostridium coccoides* group	↓*Bacteroides* MIB	↓*Enterobacteriaceae*	↓*Bifidobacterium* spp.	\	\	[45]
Diabetes	Human	\	RT‐PCR	↑*Lactobacillus* ↑*L. bugaricus* ↑*L. rhamnosum* ↑*L. acidophilus*	↓*Bifidobacteria* ↓*B. adolescentis*	\	\		\	[46]
Diabetes	Male C57BL/6 mice	60% fat (6 weeks)	16S rRNA	↑F/B ↑*Ruminococcaceae* ↑*Erysipelotrichaceae* ↑*Mogibacteriaceae* ↑*Lactobacillaceae* ↑*Oscillospira* ↑*Allobaculum* ↑*Ruminococcus* ↑*Dorea* ↑*Anaerovorax* ↑*Coprobacillus* ↑*Lactobacillus* ↓*Eubacterium dolichum*	↓*Rikenellaceae* ↓*Parabacteroides distasonis* ↑*Bacteroidaceae* ↑*Bacteroides*	↓ S24‐7 ↑*Bacteroidaceae* ↑Tannerella	↑Actinobacteria	↑Deferribacteres ↑Mucispirillum	\	[47]
Cardiovascular diseases	Human	\	Shotgun metagenomics	↓ *Eubacterium rectale*	\	\	\	\	↓ Plasma butyrate	[8]
Cardiovascular diseases	Human	\	16S rRNA	↑*Megasphaera* ↑*Oscillibacter* ↑*Faecalibacterium*	↓*Bacteroides* ↓*Prevotella*	↑*Enterobacter* ↑*Desulfovibrio*	\	\	\	[48]
Cardiovascular diseases	Human	\	Shotgun metagenomics	↓*Clostridium* ↓*Anaerostipes hadrus* ↓*Streptococcus thermophilus* ↓*Blautia* ↓SGB 4712	↑*Odoribacter splanchnicus*	↑Proteobacteria ↑*Escherichia Coli*		\	↑p‐cresol glucuronide ↑p‐cresol sulfate	[49]
Cardiovascular diseases	Male C57BL/6J mice	24% fat, 2% cholesterol (2 months)	16S rRNA	↑Firmicutes, ↑*Lactobacillus* ↑*Turicibacter* ↑*Enterococcus* ↑*Streptococcus*	↓*Muribaculaceae* ↓*Prevotellaceae*	\	\	\	\	[50]
Cardiovascular diseases	Male apoE^‐/‐^ mice	HFD (12 weeks)	16S rRNA	↓Firmicutes	↑Bacteroidetes	↑Proteobacteria	\	\	\	[51]
Cardiovascular diseases	Ldlr^‐/‐^ (Casp1^‐/‐^) mice	60% fat, 0.25% cholesterol (13 weeks)	16S rRNA	↓*Clostridium* ↓*Christensenellaceae*	↓*Odoribacter*	\	\	↓*Akkermansia*	↓Total SCFAs ↓Acetate	[52]
Gastrointestinal diseases	Human	\	RT‐PCR	↓*Roseburia hominis* ↓*Faecalibacterium prausnitzii*	\	\		\	↓SCFA, acetate, propionate	[53]
Gastrointestinal diseases	Human	\	16S rRNA	↓*Coprococcus* ↓*Phascolarctobacterium*	\	\		↑*Fusobacteriales* ↑*Fusobacteriaceae* ↓*Akkermansia*	\	[54]
Gastrointestinal diseases	C57BL/6 mice	37% fat (1 weeks)	16S rRNA	↓Firmicutes	↑Bacteroidetes	↑*Bilophila wadsworthia*	\	\	\	[55]
Gastrointestinal diseases	Human	\	16S rRNA	↓*Lactobacillus* ↑*Veillonella* ↑*Ruminococcaeae*	↑*Alistipes* ↑*Parabacteroides*	↓Proteobacteria	\	\	\	[56]
Gastrointestinal diseases	Human	\	16S rRNA	↑Firmicutes ↑*Lachnospiraceae* ↑*Ruminococcaeae*	↓Bacteroidetes	↑Proteobacteria ↑E*nterobacteriaceae*	↓Actinobacteria ↑*Eggerthella*	\	\	[57]
Gastrointestinal diseases	Human	\	16S rRNA	↑F/B ↑Firmicutes ↑*Papillibacter* ↑*Dialister* ↑*Bifidobacterium* ↑*Dorea* ↑*Blautia* ↑*Sporobacter* ↑*Actinomyces* ↑*Escherichia*	↓Bacteroidetes ↓*Bacteroidia* ↓*Odoribacter* ↓*Bacteroides* ↓*Alistipes*	\	↑Actinobacteria	\	\	[58]
Neurodegenerative diseases	Male triple transgenic mice, B6129SF2/J mice	HFD (4 months)	16S rRNA	↑*Clostridium* ↑*Mogibacteriacea* ↑*Lachnospiraceae* ↑*Enterococcaceae* ↑*Turicibacteraceae* ↓*Peptococcaceae* ↓*Dehalobacteriaceae* ↓*Lactobacillaceae*	↓S24‐7 ↑*Rikenellaceae*	\	↓*Bifidobacteriaceae*	↓RC4‐4 ↓*Bifidobacteriaceae*	\	[9]
Neurodegenerative diseases	Human	\	16S rRNA	↑*Clostridium leptum* ↓*Eubacterium ventriosum group spp*. ↓*Lachnospiraceae spp*. ↓*Marvinbryantia spp*. ↓*Monoglobus spp*. ↓*Ruminococcus torques group spp*. ↓*Christensenellaceae R‐7 spp*. ↓*Lachnospiraceae spp*. ↓*Lachnoclostridium spp*. ↓*Roseburia hominis*	\	↓*Bilophila wadsworthia*	\	\	\	[59]
Neurodegenerative diseases	Human	\	16S rRNA	↓*Faecalibacterium* ↓*Ruminococcaceae* ↓*Anaerostipes* ↓*Ruminoccocus_C* ↓*CAG‐41* ↓*F. prausnitzii*	↑*Prevotella*	\	\			[60]

Abbreviations: F/B, Firmicutes to Bacteroidetes ratio; HFD, high‐fat diet; SCFAs, Short‐chain fatty acids.

Gut microbiota modulation may be the target pathway involved in the pathogenesis of diet‐induced obesity. Cani et al. [62] also noted that obesity is closely linked to the reduction of *Bifidobacteria*. Turnbaugh et al. [63] revealed that after consuming HF/HSD, a higher proportion of the members of the *Erysipelotrichi* and *Bacilli* classes of the Firmicutes was observed compared with the control group. As an excellent model for the characterization of the human gut ecosystem, the humanized gnotobiotic mice would be beneficial as a proof of principle for “clinical trials.” Daniel et al. [40] found a decreased proportion of *Ruminococcaceae* in the HFD group, which was associated with a significant decrease in the proportion of carbohydrates, especially plant polysaccharides in the HFD formula. A nontargeted metaproteomic approach indicated that dietary transformation could change the composition of gut microbes. It could affect the metabolic pathways, such as steroid pathways [40]. Meanwhile, another study revealed that a diet with a low dietary fiber content could trigger the decrease of the Archaea kingdom, plant polysaccharides‐degrading *Prevotella*, and *Oscillospira* in HFD rats [41]. Some studies have also noted a negative correlation between gut microbiota and obesity. Kübeck et al. [42] indicated that the abundance of *Clostridium* was significantly increased in the occurrence of obesity. It also affects bile acid levels and cholesterol metabolism. This may relate to the farnesoid X receptor (FXR) signal transduction pathway of the intestinal microflora [64]. Kang et al. [43] found that high‐fat feeding could lead to an increased abundance of lipopolysaccharide (LPS)‐producing family S24‐7. It may cause the elevation of the bacterial LPS levels in systemic circulation as a feature of metabolic endotoxemia, and then lead to the aggravation of obesity with chronic low‐degree inflammation. It suggested that the increase of LPS‐producing bacteria may be treated as the potential etiology of obesity in mice fed with HFD, such as *Enterobacteriaceae* and *Desulfovibrionaceae* which were belong to Proteobacteria [44].

**Figure 1:**
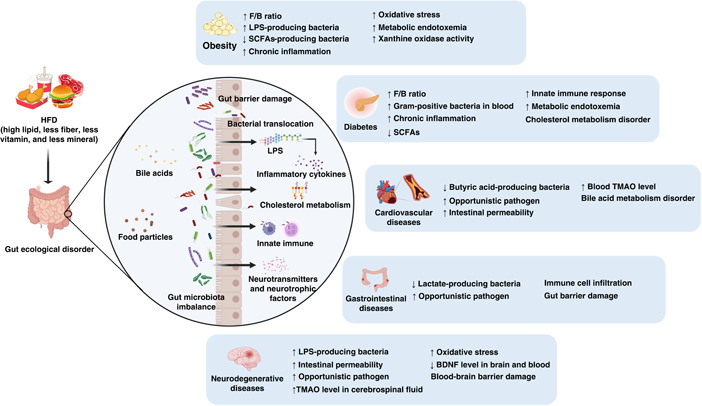
Roles of gut microbiota in chronic diseases induced by HFD. BDNF, brain‐derived neurotrophic factor; F/B ratio, ratio of Firmicutes to Bacteroidetes; HFD, high‐fat diet; LPS, lipopolysaccharide; SCFAs, Short‐chain fatty acids; TMAO, trimethylamine *N*‐oxide.

Short‐chain fatty acids (SCFAs) producing bacteria were rich in a healthy gut, including *Prevotella*, *Bacteroides, Ruminococcaceae*, and *Lachnospiraceae* [65, 66]. These SCFAs could benefit humans by maintaining the intestinal luminal anaerobic environment [44, 67]. Several studies have demonstrated that HFDs and simple sugars could reduce the abundance of SCFAs producing bacteria and increase the growth of facultative anaerobic bacteria. Additionally, Pan et al. indicated that gut microbiota is closely linked to the metabolism of purine and uric acids [68]. It is noteworthy that xanthine oxidase activity is commonly applied for analyzing the oxidative stress levels of obese people. Hence, oxidative stress levels could be considered as the critical indicators and pathways for fat tissue accumulation and hypertrophy [69]. Taken together, animal and epidemiological studies have provided the evidence that diet‐induced obesity is associated with high levels of blood glucose, lipid, and metabolic endotoxemia through influencing the composition of gut microbiota, for example, via increasing the Firmicutes to Bacteroidetes ratio, LPS‐producing bacteria, and facultative anaerobic bacteria, or reducing abundance of SCFAs producing bacteria (Table 1, Figure 1). As shown in Table 1, the influences of HFD induced obesity mainly appear in the levels of Firmicutes and Bacteroidetes phyla. However, most of the current results were evaluated using 16S ribosomal RNA (rRNA) sequencing with low accuracy at species level classification. Therefore, in‐depth studies at the genetic and functional levels of bacteria based on metagenome sequencing deems very important for the investigation of potential mechanisms among diet, obesity, and gut microbiota at species level. It is noteworthy that single‐microbe genomics shows unique insights into further strain‐level variations.

### Influences of HFD on gut microbiota toward diabetes

Diabetes is a serious chronic disease accompanied by multi‐system complications, including nephropathy, retinopathy, neuropathy, ischemic heart disease, stroke, and peripheral vascular disease [70]. The International Diabetes Federation Diabetes Atlas reported that worldwide diabetes has already reached 463 million in 2019. Amongst those, more than half of the diabetic patients were not aware that they had diabetes [71]. In 2020, WHO indicated that diabetes was already ranked in the top 10 of mortality worldwide. Type 2 diabetes (T2D) has been identified as the most prevailing form of diabetes with over 85% probability [70, 71]. Glovaci et al. [72] indicated that the main causes of T2D may be attributed to the deficiency of insulin secretion, which could be triggered by ethnicity, family history, age, unhealthy diet, smoking, and obesity.

It is noteworthy that HFD is the main unhealthy dietary pattern attributed to diabetes. Several studies have proven that intestinal bacteria play important roles toward HFD induced diabetes. Cani et al. [45] revealed that gut *Bifidobacterium spp*. exhibited a negative correlation to endotoxemia in HF mice. Recovery of *Bifidobacterium spp*. could be accompanied by improvement of glucose‐induced insulin secretion, glucose tolerance, and inflammation reduction. This result was confirmed by Lê et al. [46] reporting that the abundance of *Bifidobacteria* significantly decreased in diabetic patients, suggesting that the abundance of *Bifidobacteria* could be served as a microbial biomarker for diabetes [46]. An epidemiological study also found that diabetic patients would accompany a high abundance of gram‐positive anaerobic bacteria in blood [73]. At the same time, another study showed that >90% of bacteria belonging to the gram‐negative Proteobacteria phylum in the blood of patients with type 2 diabetes [74]. The data indicated that diabetes may affect gut permeability, further leading to the leakage of bacteria in the blood and harm to human health. Sato et al. [73] also reported that the abundance of facultative anaerobic bacteria, such as total *Lactobacillus*, in diabetic groups were significantly increased in fecal samples, which is similar to the changes observed in obesity. Hence, *Lactobacillus* of facultative anaerobic bacteria may be considered as the potential target for the “Bad” gut microbiota triggered by HFD induced diabetes or obesity. However, Forslund et al. reported that the abundance of *Lactobacillus* is normal in T2D patients in Sweden or Denmark [75]. Nevertheless, the composition of gut microbiota could just be a dynamic reference that deserved a further investigation.

**Figure 2:**
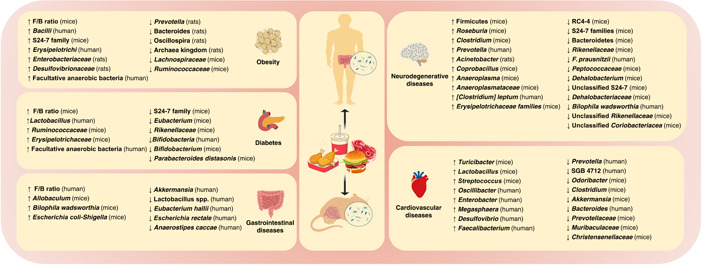
Influence of intestinal microbiota in chronic diseases under HFD model. HFD, high‐fat diet.

Microbial metabolites derived from an imbalance of “Good” and “Bad” gut microbiota are also linked to HFD‐induced diabetes. Wang et al. [76] found that an imbalance of “Good” and “Bad” gut microbiota led to the attenuation of the bacterial metabolite SCFAs and activated HFD‐Gut microbiota‐Butyrate‐Insulin resistance pathway in HFD‐induced diabetes. The similar results were also noted by Qin et al. and Karlsson et al. [77, 78]. Liu et al. [47] also found that HFD could significantly change the composition of gut microbiota and related metabolic pathways in T2D mice, where the abundance of S24‐7 family, *Rikenellaceae, Parabacteroides distasonis* (*P. distasonis*), and *Eubacterium dolichum* (*E. dolichum*) were significantly decreased [47] (Figure 2, Table 1). Early studies have already confirmed that the mentioned gut microbiota above possessed health benefits. For example, Weng et al. [79] indicated that *P. distasonis* can produce succinic acid and secondary bile acids, and participate in host metabolism regulation. Turnbaugh et al. [10] revealed that *the E. dolichum* can degrade fructose‐containing carbohydrates to promote the production of SCFAs, while *Rikenellaceae* was related to inflammatory inhibition. These findings all confirmed that gut microbiota possesses numerous functional impacts toward human health and plays an important role in the progress of diabetes.

Diabetes is well known to be closely correlated to the prevalence of obesity. As shown in Figure 1, the influences of the composition of gut microbiota and the potential mechanism of action exhibit highly commonality in obesity and diabetes. Raising evidence revealed that the increased F/B ratio, metabolic endotoxemia, and chronic inflammation are harmful to human health. Meanwhile, it suggests that SCFAs could have health benefits against obesity and diabetes. For details, the influences of the abundance of gut microbiota also exhibit uniformity in obesity and diabetes shown as Figure 2, such as the increase of *Erysipelotrichaceae* and facultative anaerobic bacteria. Present results suggested that *Erysipelotrichaceae* and facultative anaerobic bacteria could be identified as the “Bad” bacteria triggered the incidences of obesity and diabetes. However, it requires further confirmation.

### Influences of HFD on gut microbiota toward CVDs

CVDs include coronary heart disease, stroke, heart failure, and peripheral artery disease [80]. CVDs are the primary cause of global mortality, accounting for 32% of global deaths in 2019. More than three‐quarters of cardiovascular deaths occur in low‐ and middle‐income countries[81]. In China, 40% deaths are caused by CVDs [82]. Hypertension, atherosclerotic plaque formation, myocardial infarction, and heart failure are highly related to gut microbiota [28, 83].

Polsinelli et al. and Tang et al. [84, 85] reported that patients with injured intestinal epithelial barrier also accompany heart failure. Tang et al. reported that heart failure is also highly related to specific intestinal microbes, including an increase of *Escherichia coli, Klebsiella pneumoniae*, and *Streptococcus viridans*. It was further proved that gut microbiota and its metabolites may enter the blood circulation, and then invade the organs [86]. In addition, Kim et al. [8] also proved that hypertension will lead to a significant decrease of abundances of butyric acid‐producing bacteria *Eubacterium rectale*. Yin et al. found that patients with stroke or transient ischemic attack have significantly reduced the abundance of *Bacteroides, Prevotella*, and *Faecalibacterium*, but enriched opportunistic pathogens such as *Enterobacter, Megasphaera, Oscillibacter*, and *Desulfovibrio* [48]. In the impaired metabolic status and drug treatment, the depletion of unknown *Clostridiaceae* (SGB 4712) has been associated with major metabolites of *p*‐cresol and higher abundances of Proteobacteria was found in patients with acute coronary syndrome than health individuals [49, 87]. Taken together, increasing evidence suggests that onset and progress of CVDs could affect the abundance of specific microbes and lead to gut microbiota dysbiosis. However, the potential mechanisms of influencing gut microbiota toward CVDs should be clarified.

Hypertension and hypercholesterolemia are the main risk factors for CVDs. HFD was treated as the main cause of hypertension and hypercholesterolemia toward CVDs. It is largely responsible for hyperlipidaemia, liver steatosis, and gut microbiota dysbiosis [88]. Zhang et al. [50] proven that HFD may promote myocardial injury through increasing intestinal permeability and the elevation of serum trimethylamine *N*‐oxide (TMAO) levels. Shi et al. [51] also noted that HFD can induce atherosclerosis with endothelial dysfunction caused by TMAO. TMAO is an oxidation product metabolized by the metabolite trimethylamine of intestinal microbial through liver flavin‐containing monooxygenase 3 (FMO3) [89]. Studies have confirmed that TMAO can be identified as an adverse contributor to CVDs [90, 91, 92]. As shown in Figure 1, apart from intestinal permeability and TMAO levels, bile acids are also a potential factor involved in the mechanism of gut microbiota toward CVDs. Multiomics analysis revealed that *Lachnoclostridium* and unidentified *Enterobacteriaceae* were significantly enriched in CVDs. It also found that they were closely linked with taurocholic acid, participated in the bile acid metabolism [93]. Primary bile acids can be transformed into secondary bile acids with the help of gut microbiota, including taurocholic acid. As a signal molecule, bile acids could bind with FXR and Takeda G protein‐coupled receptor 5 (TGR5) to further promote immune cell filtration and regulate lipid metabolism. However, excess bile acids may contribute to inflammation and cholesterol metabolism disorder [94]. In Ldlr^−/−^ (Casp1^−/−^) mice with pro‐inflammatory microorganisms exposed to HFD, the systemic inflammation was accompanied with the changed composition of intestinal flora, decreased SCFAs level, but non affected TMAO and gut integrity, which eventually accelerated atherosclerosis [52]. The present findings established that HFD induced CVDs may lead to gut microbiota dysbiosis by disrupting the metabolism of bile acid, TMAO and SCFAs produced by butyric acid producing bacteria, opportunistic pathogens, *Lachnoclostridium*, *unidentified Enterobacteriaceae*, and SCFAs producing bacteria (Table 1).

### Influences of HFD on gut microbiota toward gastrointestinal diseases

Gut microbiota mainly reside in gastrointestinal tract, playing critical roles in gastrointestinal diseases. Richard and Sokol indicated that a functional disturbance of the gut ecosystem is highly associated with gastrointestinal diseases [95]. Ulcerative colitis (UC) and irritable bowel syndrome (IBS) are common chronic diseases of the gastrointestinal tract with characterization of the repeated attacks and long‐term illness [96]. UC is defined as the progressive or chronic remittent inflammatory condition known as inflammatory bowel disease (IBD), mainly distributed in the descending colon, sigmoid colon or rectum [97]. Bloody diarrhea, abdominal pain, and rectal urgency are the typical symptoms of UC [98]. Rashvand et al. [99] conducted on newly diagnosed UC patients (<6 months), found that the number of patients with high fat intake was significantly higher than control group. Furthermore, Jowett et al. [100] also pointed out that the high intake of meat is related to an increased risk of recurrence of colitis. Ma et al., and Teixeira et al. also revealed that HFD could effectively aggravate the progress of UC [101, 102]. Besides, Rashvand et al. has noted a close correlation between saturated fat and UC, while high intake of total polyunsaturated fatty acids or monounsaturated fatty acids was linked with high risk of UC [99]. Studies from the United States, Spain, Ireland, and the Czech Republic also showed that UC patients appeared with a significantly decrease of the abundance of *Akkermansia* [55, 103, 104]. However, numerous research reported the abundance of *Escherichia rectale* was dominant decline in UC [54, 105, 106]. Devkota et al. revealed that milk derived fat could significantly promote the proliferation of pathogenic bacterium *B. wadsworthia* by boosting the change of bile acid composition to exacerbate the onset of colitis [55]. Furthermore, Devkota et al. found that under the protection of bile, *B. wadsworthia* in the GF IL10^−/−^ mice would establish a niche containing other pathogenic bacteria or pathogens [55]. In addition, metabolic products such as H_2_S and secondary bile acids can destroy the intestinal barrier and increase the infiltration of immune cells, leading to an increase in the prevalence of IBD. Li et al. proved that UC with HF/HSD will significantly increase the abundance of *Allobaculum* and *Escherichia coli‐Shigella* [107].

IBS is one of the most common gastrointestinal diseases, characterized as abdominal pain and transformation in bowel habits, with a prevalence rate ranging from 5% to 18% [108]. IBS is mainly divided into diarrhea type and constipation type, both of which are related to gut microbiota [33]. The gut microbiota are affected by environmental factors like diet patterns, stress infection, antibiotic use, and host factors such as immune activation and low‐grade inflammation [96]. The changes in intestinal bacteria in patients with diarrhea‐predominant IBS are mainly related to the decrease of beneficial bacteria, such as *Lactobacillus spp*., Actinobacteria, and Bacteroidetes, while significantly increasing the harmful bacteria such as Proteobacteria [56–58]. In contrast, constipation‐predominant IBS patients have significantly increased Firmicutes and decreased lactate‐producing bacteria like *Eubacterium hallii* and *Anaerostipes caccae* [109, 110]. Furthermore, fecal bacteria from IBS patients were colonized in rodents, leading to the appearance of IBS‐like symptoms, indicated that microbes played an undeniable role in the occurrence and development of IBS [111, 112]. The etiology of IBS may be associated with abnormal gastrointestinal motility, increased visceral sensitivity, low‐grade inflammatory reaction, and brain‐gut axis caused by intestinal bacteria disorder [113]. Diet is one of the factors directly affecting intestinal bacteria. A study of 197 IBS patients showed that the course of the disease was related to eating high‐fat or fried foods in more than half of patients [114]. In conclusion, HFD may promote the development of UC and IBS through intestinal bacteria (Figure 1, Table 1). However, the corresponding mechanism of HFD induced UC and IBS by gut microbiota modulation remains obscure and is worthy of further exploration.

### Influences of HFD on gut microbiota toward neurodegenerative diseases

Physical functions of the human body will gradually deteriorate with age. Feigin et al. [115] indicated that cognitive impairment or dementia with deterioration in memory, thinking, behavior, and self‐care ability are the main reasons for the worldwide disability of elderly. Due to the aging population and environmental factors, the epidemic is increasing at an alarming rate. There are about 50 million dementia patients worldwide, and nearly 10 million new cases increase every year [116]. Alzheimer's disease (AD) with clinical manifestations of memory loss and cognitive impairment are the most common form of dementia which is almost incurable [32, 50].

Microbes may be potential candidates for biotherapy intervention in AD. Compared with the control, decreased *Faecalibacterium Prausnitzii (F. Prausnitzii)* in feces was associated with lower cognitive score [60]. Further, isolated *F. Prausnitzii* could improve the cognitive impairment of AD mice [60]. In addition, a cohort by Verhaar et al. [59], conducted on 170 AD dementia patients from the Netherlands, found that the low abundance of SCFAs producing bacteria was positively correlated with incidence of dementia biomarkers such as positive amyloid and p‐tau status using 16S rRNA sequencing and machine learning models.

As shown in Table 1, HFD could promote the onset of AD by impacting the gut microbiome. In mouse models with a genetic predisposition to AD, there were similarities between HFD feeding and genetically predisposed AD mice, and this effect was superimposed [9]. Heatmap showed that the increase in Firmicutes phylum, *Anaeroplasmataceae*, *Erysipelotrichaceae* families, *Coprobacillus*, *Clostridium*, *Anaeroplasma* and *Roseburia* genera as well as the decrease in Bacteroidetes phylum, *Peptococcaceae*, *Rikenellaceae*, *Dehalobacteriaceae*, S24‐7 families, RC4‐4, *Dehalobacterium*, *Unclassified Coriobacteriacea*, and *Unclassified Rikenellaceae* were largely relevant to brain hypometabolism.

In parallel, the results demonstrated that abnormal specific intestinal bacteria and their metabolites might occur earlier than the appearance of obvious cognitive impairment, which could be further studied as a predictive marker. High‐fat feeding resulted in the imbalance of intestinal bacteria exhibited an increase in the number of *Acinetobacter*‐producing LPS [117]. Wei et al. demonstrated that the outer membrane containing LPS injections could cause cognitive impairment, mainly through increasing barrier permeability, activating glycogen synthase kinase‐3*β* (GSK3*β*), tau hyperphosphorylation, and activating microglia, which will lead to neuroinflammation [118]. In addition, a long‐term HFD may destroy the blood‐brain barrier and allow LPS to reach the brain regions related to AD, such as the neocortex and hippocampus [119]. In the microglia membrane, an LPS‐CD14 complex combined with LPS and CD14 further interacted with toll‐like receptor 4 (TLR‐4). Then, TLR‐4 activated astrocytes and released inflammatory mediators to induce oxidative stress and inflammation, finally resulting in the accumulation of *β*‐like protein and intraneuronal neurofibrillary tangles (NFT) in the brain [117]. In addition, the accumulation of LPS near the nucleus of neurons led to changes in the expression of genes encoding various proteins, such as synapsin‐1, which was also one of the possible mechanisms. Pathogens themselves could also cross the damaged intestinal barrier, shuttle through the systemic circulation system and trigger inflammation, and might enter the central nervous system to participate in the activation of microglia [120]. Neurotransmitters were closely related to cognitive function. The majority of neurotransmitters could be produced by gut microbiota, including gamma‐aminobutyric acid (GABA) (*Lactobacillus* and *Bifidobacterium)*, serotonin and dopamine (*Escherichia coli*), and acetylcholine (*Lactobacillus*) [121]. These neurotransmitters regulated the expression of neurotrophic factors such as brain‐derived neurotrophic factor (BDNF) secreted by intestinal smooth muscle cells [122]. The low expression of BDNF in the brain and plasma of HFD animals was accompanied by intestinal bacteria disbalance [122]. A high saturated fat diet led to the increase of TMAO, a gut microbial metabolite [123]. High levels of TMAO in the cerebrospinal fluids of patients with mild cognitive impairment and Alzheimer's disease might be related to the pathology and neurodegeneration of tau [124]. Thus, TMAO may also promote the development of cognitive impairment.

Therefore, the elderly should pay special attention to a healthy diet. HFD increases the risk of cognitive impairment and even aggravates AD (Figures 1 and 2). Notably, most existing studies focus on the role of intestinal bacteria in cognitive impairment caused by HFD. However, there are few studies reporting how HFD promotes disease development through intestinal bacteria via the AD model.

### Interacted mechanisms of HFD on gut microbiota toward chronic diseases

As mentioned above, numerous studies noted that HFD is closely related to gut microbiota dysbiosis in chronic diseases. The corresponding impacts on the gut microbiota toward obesity, diabetes, gastrointestinal diseases, neurodegenerative diseases, and CVDs are summarized in Table 1, Figures 1 and 2. Further interaction mechanisms toward HFD induced chronic diseases through gut microbiota modulation underlying bile acids, LPS, SCFAs, and TMAO were demonstrated as followed.

#### Bile acid

**Figure 3:**
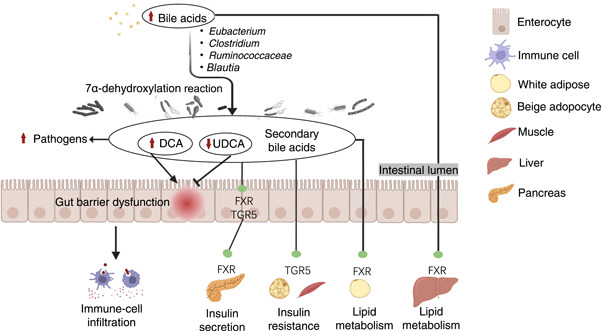
Potential mechanisms of interaction between HFD and chronic diseases via bile acid. Cholesterol can stimulate the secretion of bile acids, which forms a secondary bile acid with the help of intestinal bacteria through a 7α‐dehydroxylation reaction. Secondary bile acids like DCA with high hydrophobicity disrupt the plasma membrane structure and destroy the intestinal barrier, while opportunistic pathogens resistant to bile rapidly multiply. As a signal molecule, bile acids bind FXR and TGR5 to further promote immune‐cell infiltration, regulate insulin resistance and regulate lipid metabolism. DCA, deoxycholic acid; FXR, farnesoid X receptor; TGR5, Takeda G protein‐coupled receptor 5; UDCA, ursodeoxycholic acid.

Bile acids are critical to fat metabolism, existing in the systems of enterohepatic circulation. Staley et al. and Lin et al. indicated that specific intestinal bacteria would interact with bile acids to affect the levels of secondary bile acids by 7α‐dehydroxylation reaction, such as *Eubacterium*, *Clostridium, Ruminococcaceae*, and *Blautia* [125, 126]. Lin et al. [126] have demonstrated that HFD could significantly increase the expression levels of deoxycholic acid (DCA), while decreasing the levels of ursodeoxycholic acid (UDCA). The elevation of DCA levels can disrupt the membrane structure of plasma and destroy the membrane integrity [126], whereas the descending UDCA levels can lead to the intestinal barrier dysfunction [127, 128]. The increase in DCA levels and the decrease of UDCA levels will also promote the growth of pathogens and accompany the production of hydrogen sulfide which will injure the intestinal wall. As shown in Figure 3, bile acids may mainly modulate the lipid metabolism, insulin resistance, and immune cell infiltration through regulating TGF5 or FXR receptors mediated signaling [129]. Altogether, high fat intake may trigger chronic diseases by simulating the secretion of bile acids through various mechanisms. Lin et al. and Parséus et al. indicated that cholic acid (CA), chenodeoxycholic acid (CDCA), lithocholic acid (LCA), and DCA are identified as the agonists of FXR receptors, while UDCA and T*β*MCA are treated as the antagonists of FXR [64, 126]. Indeed, several studies also found that the inhibition of FXR receptor and the activation of TGR5 mediated signaling are closely linked with the lipid metabolism regulation, treated as the interacted mechanisms of bile acids in HFD induced chronic diseases [64, 126, 129].

#### LPS

**Figure 4:**
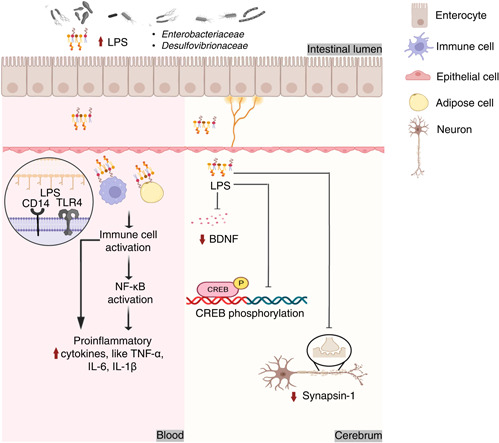
Potential mechanisms of interaction between HFD and chronic diseases via LPS. LPS, a structural component of the outer membrane of gram‐negative bacteria, penetrate the intestinal wall and reaches the corresponding tissues such as the blood systemic circulation and brain. TLR4 is expressed in immune cells, adipocytes, glial cells, and other cells. Hence, LPS causes inflammation via NF‐κB, thus promoting obesity and brain degeneration. Furthermore, LPS inhibits the expression of BDNF and CREB phosphorylation, and inhibits the expression level of synapsin‐1 in the brain. BDNF, brain derived neurotrophic factor; CD14, cluster of differentiation 14; CREB, cAMP response element‐binding protein; IL‐6, interleukin 6; IL‐1*β*, interleukin 1*β*; LPS, lipopolysaccharide; NF‐κB, nuclear factor κB; TLR4, Toll‐like receptor 4; TNF‐*α*, tumor necrosis factor α.

LPS is the vital structural component of the membrane of gram‐negative bacteria. As mentioned above, evidence indicated that HFD could induce chronic diseases accompanying with the elevation of bacterial LPS levels in systemic circulation [43]. Some LPS‐producing bacteria could be the potential etiology of HFD‐induced chronic diseases, such as *Enterobacteriaceae* and *Desulfovibrionaceae* [44]. As shown in Figure 4, bacterial LPS could effectively activate receptors CD14 and TLR4 on surfaces of immune cells, adipocytes, glial cells [129]. Zakaria et al. and Jeong et al. also proved that LPS could stimulate the secretion of pro‐inflammatory cytokines through the activation of nuclear factor kappa‐B (NF‐κB) pathway, including Tumor necrosis factor alpha (TNF‐*α*), Interleukin 6 (IL‐6), and Interleukin 1 beta (IL‐1*β*) [117, 130]. Additionally, HFD will increase the intestinal permeability which will lead to the diffusion of LPS in the systemic circulation [131]. It also will lead to the reduction of cognitive ability and the aggravation of anxiety through blood‐brain barrier disruption by inhibiting the expression levels of BDNF and the phosphorylation levels of cAMP response element‐binding protein (CREB) [130, 132]. Additionally, Zakaria et al. and Jeong et al. also revealed that LPS could affect the expression of synapsin‐1 protein gene, which is closely linked to the HFD triggered psychiatric disorders [117, 130]. Current findings established that HFD may trigger chronic diseases mainly through activation of the TLR4/NF‐κB pathway or inhibiting the expression levels of BDNF, CREB, and synapsin‐1 by LPS stimulation. However, the interacted mechanism among blood‐brain barrier, gut‐brain axes, and gut immune barrier remain a diagnostic challenge to scientists.

#### SCFAs

**Figure 5:**
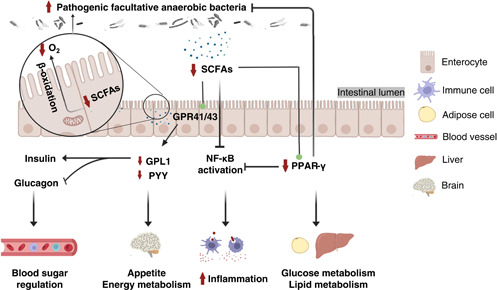
Potential mechanisms of interaction between HFD and chronic diseases through SCFAs. HFD reduces the synthesis of SCFAs in the host. SCFAs can inhibit an inflammatory reaction, maintain the anaerobic environment of the intestinal cavity, and regulate appetite, energy metabolism, glucose and lipid metabolism. GPL1, glucagon‐like peptide‐1; GPR, G protein‐coupled receptor; NF‐κB, nuclear factor κB; PPAR‐*γ*, peroxisome proliferator‐activated receptor *γ*; PPY, peptide YY; SCFAs, short‐chain fatty acids.

Acetate, propionate, and butyrate are the metabolic products of anaerobic fermentation of dietary fiber by colon bacteria, which can be used as signal molecules to further regulate host metabolic homeostasis. Generally, SCFAs, especially butyrate, increase the expression of glucagon‐like peptide‐1 (GPL1) and peptide YY (PYY) by activating G protein‐coupled receptors (GPRs) and peroxisome proliferator‐activated receptor gamma (PPAR‐*γ*). The satiety hormones GPL1 and PYY further act on the brain‐gut axis to regulate appetite and energy metabolism [133, 134]. PPAR‐*γ* can maintain an intestinal anaerobic environment (related to *β*‐oxidation), activate glucose and lipid metabolism genes, and inhibit inflammatory response, related to the inhibition of NF‐κB [135]. Moreover, butyrate acts on colonocytes, promotes *β*‐oxidation of mitochondria, and reduces oxygen saturation in the intestinal cavity [136]. HFD greatly reduces the content of SCFAs, resulting in energy metabolism disorder and excessive reproduction of the pathogenic facultative anaerobic bacteria *Escherichia coli* [137] and the production of pro‐inflammatory factors [33] (Figure 5).

#### TMAO

**Figure 6:**
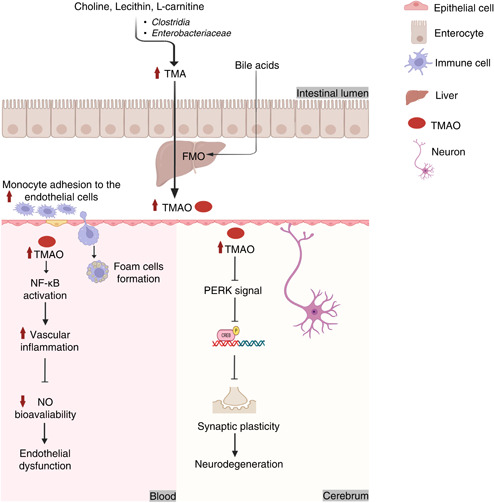
Potential mechanisms of interaction between HFD and chronic diseases through TMAO. Choline can be metabolized into TMA by intestinal bacteria, which is further processed into TMAO by FMO in the liver. In addition, the expression of FMO is also affected by bile. TMAO entering the blood vessels is related to vascular inflammation, endothelial dysfunction, foam cell formation, atherogenic plaques as well as insulin resistance. TMAO in the brain can affect synaptic plasticity and cause neurodegeneration, and the atherosclerosis of brain blood vessels is also an important factor of dementia. CREB, cAMP response element‐binding protein; FMO, flavin‐containing monooxygenase; HFD, high‐fat diet; NF‐κB, nuclear factor κB; NO, nitric oxide; PERK, protein kinase RNA‐like ER kinase; TMA, trimethylamine; TMAO, trimethylamine *N*‐oxide.

Numerous studies have implied that TMAO could be an independent risk factor for CVDs [138, 139]. Vogt et al. [124] also noted a positive correlation between high TMAO levels in the brain and the deterioration of cognitive impairment and AD. As mentioned above, HFD can significantly promote the production of choline and modulate the abundance of specific gut microbes. Additionally, methylamine‐contained nutrients are commonly existed in fat‐rich western diets, such as choline, lecithin, and *L*‐carnitine [138]. The fact that long‐term consumption of western diets will greatly increase the risk of CVDs and nervous system diseases suggests that HFD possessed a close link to  CVDs perhaps through the high levels of choline. As shown in Figure 6, diet‐induced choline could be further metabolized by *Clostridia* and *Enterobacteriaceae* and converted into trimethylamine (TMA), followed by the concomitant conversion into TMAO by Flavin‐containing monooxygenase (FMO) in liver [140, 141, 142]. Hence, HFD can effectively modulate TMAO. It is noteworthy that the expression levels of FMO also could be affected by the regulation of HFD mediated bile acids [143]. The increase of TMAO will further stimulate the secretion of inflammatory cytokines through the activation of NF‐κB pathway, leading to the endothelial dysfunction. Apart from that, the increase of TMAO also will aggravate neurodegeneration through attenuating the synaptic plasticity by protein kinase RNA‐like ER kinase (PERK) signaling inhibition. Altogether, present results demonstrated that HFD will induce CVD through the activation NF‐κB pathway or inhibition of PERK signaling by the increase of TMAO. However, whether TMAO also plays an important role in the mechanism of other chronic diseases deserves further exploration.

## CONCLUSION

Chronic diseases are the leading cause of death in the world. HFD has been noted as the main risk factor for chronic diseases. It may lead to gut microbiota dysbiosis, which will impact the aggravation of chronic diseases. In this review, the influences of HFD on the composition of gut microbiota and the potential mechanisms toward corresponding chronic diseases are systematically summarized. First, the review revealed that HFD induced obesity and diabetes exhibited high commonality and features on the influence of the composition of gut microbiota, accompanied by the increase of F/B ratio, metabolic endotoxemia, and chronic inflammation. It also suggested that *Erysipelotrichaceae, Bifidobacteria* and facultative anaerobic bacteria could be identified as bacterial markers to trigger incidences of obesity and diabetes. *Enterobacteriaceae* and *Desulfovibrionaceae* may be the potential etiology of obesity, while *P. distasonis* and *E. dolichum* are closely related to diabetes. Second, this review also established that butyric acid producing bacteria, opportunistic pathogen, *Lachnoclostridium*, and *unidentified Enterobacteriaceae* are identified as the characteristic microbes in HFD induced CVDs. Third, lactate‐producing bacteria and opportunistic pathogens are treated as the common microbial markers in HFD‐induced gastrointestinal diseases with details as shown in Figure 2 and Table 1, including Proteobacteria, *Escherichia coli‐Shigella*, and *Allobaculum*. Fourthly, LPS‐producing bacteria and opportunistic pathogens were also identified as the general microbial markers in HFD induced neurodegenerative diseases, including *E. coli*, *Desulfovibrio*, and *unidentified Enterobacteriaceae*.

Additionally, this review also discussed that bile acids, LPS, SCFAs, and TMAO could be the commonalities and features of bacterial metabolites in chronic diseases. Further interacted mechanisms toward HFD induced chronic diseases through gut microbiota modulation underlying bile acids, LPS, SCFAs, and TMAO were illustrated in Figures 3, 4, 5, 6. As shown in Figures 3, 4, 5, 6, accumulating evidence concluded that HFD may influence the gut microbiota mediated bile acids and chronic diseases through the inhibition of FXR receptor and activation of TGR5 mediated signaling. HFD can mainly affect gut microbiota mediated SCFAs to induce chronic disease through activation of the NF‐κB pathway and PPAR‐*γ* inhibition. Numerous results also established that microbes mediated LPS could have a positive impact on the promotion of HFD induced chronic diseases through the activation of the TLR4/NF‐κB pathway, and inhibition of the expression levels of BDNF, CREB, and synapsin‐1. TMAO can be considered as an independent risk factor for CVDs. HFD may influence CVDs by the modulation of gut microbiota interacted TMAO through inhibiting PERK signaling. However, the impact of TMAO toward other chronic diseases remains scarce. While current results are based upon 16S rRNA sequencing with low accuracy at species level classification, future studies based on metagenome sequencing to investigate potential mechanisms among diet, obesity, and gut microbiota at the genetic and functional levels of bacteria remain a prospective challenge to scientists. It is noteworthy that single‐microbe genomics shows unique insights into further strain‐level variations.

Interactive mechanisms of gut microbiota modulation in the human body are very complex, especially given the impacts of diet on chronic diseases. This review systematically summarizes the influences of gut microbiota and their corresponding bacterial metabolites, which we hope will provide new insights into mechanisms among microbiota, metabolites, and immune responses during chronic diseases. The suggestion of potential biomarkers may improve holistic thinking about the issues surrounding long‐term care and disease management.

## AUTHOR CONTRIBUTIONS

*Conceptualization*: Jiali Chen. *Methodology*: Jiali Chen and Weibin Bai. *Software*: Jiali Chen and Yuhang Xiao. *Validation*: Jiali Chen, Yuhang Xiao, and Weibin Bai. *Formal analysis*: Jiali Chen and Yuhang Xiao. *Investigation*: Jiali Chen and Yuhang Xiao. *Resources*: Jiali Chen and Weibin Bai. *Data curation*: Jiali Chen, Yuhang Xiao, and Weibin Bai. *Writing—original draft preparation*: Jiali Chen and Yuhang Xiao. *Writing—review and editing*: Dongmei Li, Shiqing Zhang, Yingzi Wu, Qing Zhang, Jiali Chen, and Weibin Bai. *Visualization*: Jiali Chen, Dongmei Li, and Yuhang Xiao. *Supervision*: Jiali Chen and Weibin Bai. *Project administration*: Jiali Chen and Weibin Bai. *Funding acquisition*: Jiali Chen and Weibin Bai. All authors have read and agreed to the published version of the manuscript.

## CONFLICT OF INTEREST

The authors declare no conflict of interest.

## Data Availability

Data sharing is not applicable to this article as no new data were created or analyzed in this study. No new data and script were used in this paper. Supplementary materials (figures, tables, scripts, graphical abstracts, slides, videos, Chinese translated versions and updated materials) may be found in the online DOI or iMeta Science http://www.imeta.science/.
